# Laccase mediated delignification of pineapple leaf waste: an ecofriendly sustainable attempt towards valorization

**DOI:** 10.1186/s13065-019-0576-9

**Published:** 2019-04-23

**Authors:** Rintu Banerjee, Anjani Devi Chintagunta, Subhabrata Ray

**Affiliations:** 1 Agricultural & Food Engineering Department, Indian Institute of Technology, Kharagpur, West Bengal 721302 India; 2 Advanced Technology Development Centre, Indian Institute of Technology, Kharagpur, West Bengal 721302 India; 3 Chemical Engineering Department, Indian Institute of Technology, Kharagpur, West Bengal 721302 India

**Keywords:** Cellulose, Enzymatic pretreatment, Laccase, Pineapple leaf waste

## Abstract

**Background:**

Escalating energy security, burgeoning population and rising costs of fossil fuels have focussed our attention on tapping renewable energy sources. As the utilization of food crops for biofuel production culminates into food vs. fuel dilemma, there is an intensive need for alternatives. Production of biofuels from lignocellulosic biomass owing to its profuse availability and high holocellulose content is a promising area for research.

**Results:**

In the present study, pineapple leaf, an agro-industrial waste was pretreated with laccase to enhance the enzymatic digestibility of the substrate for improved production of reducing sugar. Variables determining enzymatic delignification of pineapple leaf waste have been optimized by response surface methodology based on central composite design. Maximum delignification of 78.57%(w/w) resulted in reducing sugar of 492.33 ± 3.1 mg/g in 5.30 h. The structural changes in pineapple leaf waste, after laccase treatment, were studied through Fourier transformed infrared spectroscopy, X-ray diffraction and Scanning electron microscopy. Specific surface area, pore volume, and pore diameter of the substrate were studied using the Brunauer–Emmett–Teller and Barrett–Joyner–Halenda methods and found a significant increase in the aforementioned parameters after delignification.

**Conclusion:**

Laccase mediated delignification of pineapple leaf waste is a cleaner sustainable process for enhanced production of reducing sugar which can accomplish the demand for biofuels.
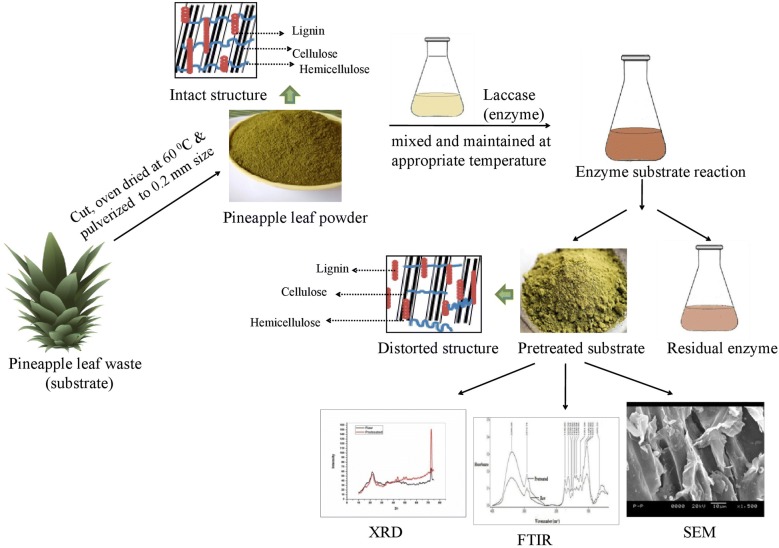

## Introduction

Lignocellulosic biomass is a potential renewable resource for biofuel production due to wide availability, low cost and rich holocellulose content. Predominant lignocellulosics that have been employed for bioenergy production are oil palm frond [1], poplar [2], switch grass [3] and barley straw [4]. In addition to these feedstocks, pineapple waste is another prominent lignocellulosics, which is being produced abundantly by canning industries throughout the world. The worldwide pineapple production was estimated to be 24.80 Mt from 1.02 Mha for the year 2013–2014 [5] the production from India being 1.74 Mt from 0.11 Mha [6]. A major share of the total production is processed in canneries resulting in generation of substantial amount of waste comprising the peel, crown, leaves etc. The peel and stems of pineapple waste is employed for the production of valuable products like bromelain and vinegar [7]. A part of leaf waste is used in making rope fibres and paper industries. Being rich in reducing sugar and holocellulosic content (65–67%, w/w), pineapple leaf waste has a great potential for bioethanol production.

A major impediment in the utilization of lignocellulosic biomass as a substrate for ethanol production is the presence of lignin that resists chemical or enzymatic digestion of cellulose and hemicellulose polymers [8]. Lignin and hemicelluloses form a barrier around the crystalline and amorphous regions of cellulose. Hence, the breakdown of lignin is essential for accessing the cellulose component of the lignocellulosics, which requires an appropriate pretreatment process. Among the various methods of pretreatment like physical, chemical, physicochemical and biological, enzymatic pretreatment or delignification process is beneficial owing to its high specificity, low energy requirements, higher yield of reducing sugar and minimum by-product formation.

The mechanism involved in various pretreatment processes specifically pertaining to the alterations in physical structure and chemical composition of biomass should be understood completely. The ultimate goal of any pretreatment process is to enhance the accessibility of biomass to the enzymes for efficient generation of reducing sugar at low enzyme dose. The conventional pretreatment methods result in the formation of furfurals, hydroxymethyl furfurals, acetic acid and formic acid etc., that inhibit the subsequent steps of bioethanol production. During enzymatic delignification, enzyme acts specifically and release phenolic intermediates which act as natural mediators enhancing the delignification process [9]. High yield of reducing sugar was reported during saccharification after chemical pretreatment of Kans grass [10]. It was reported that enzymatically delignified lignocellulosics yielded high reducing sugar content after saccharification when compared to that treated with steam explosion and acid pretreatments [11, 12].

Owing to the aforementioned advantages of enzymatic pretreatment, laccase mediated delignification of pineapple leaf waste was carried out in the present study. Laccase is an extracellular, multi-copper enzyme which degrades lignin through molecular oxygen and oxidizes different aromatic and non-aromatic compounds present in lignin [13]. Till date, very few reports are available on the enzymatic pretreatment of different lignocellulosics and its effect on reducing sugar production [14, 15].

Recently, multivariate statistical techniques are predominantly being used for optimization of process parameters. Factorial designs viz., central composite and Box–Behnken are used, for evaluating the effect of one factor at several levels of other factors in addition to the interaction among them, which is not possible in one-factor-at-a-time [16]. In the present work, optimization of enzymatic pretreatment of pineapple leaf waste was carried out by response surface methodology (RSM) based on central composite design (CCD). It is worth mentioning that decrease in lignin and hemicellulose content due to pretreatment process affects the physical properties of the cellulosic component such as crystallinity, surface area of the substrate etc. Structural characterization was done to corroborate the delignification process and porosity measurements to assess the accessible surface area of the substrate to cellulase for reducing sugar production. Enzymatic delignification is a green, sustainable and eco-friendly approach for production of biofuel and other bio-products.

## Methods

### Substrate and its biochemical characterisation

Pineapple (*Ananas comosus*, Giant Kew variety) leaf waste was collected locally from the farm of IIT Kharagpur, India. It was chopped, sun dried, pulverised to particle size of 0.2 mm and used in the present study. Lignin, cellulose and hemicellulose content of raw and delignified pineapple leaf wastes were estimated using standard protocols [17–19]. The amount of reducing sugar was measured by dinitrosalicylic acid method (DNS) [20]. The elemental analysis of moisture free raw and delignified pineapple leaf waste was done by the CHNS analyser (M/s Elementar, VarioMicrocube, Germany).

### Enzyme

Enzymatic delignification of the substrate was done using laccase from *Pleurotus djamor*. Enzyme activity was determined spectrophotometrically using 2,2′-azino-bis (3-ethylbenzothiazoline-6-sulphonic acid) (ABTS) as substrate [21]. The activity of laccase was represented in international unit (IU) which is defined as the amount of enzyme required to oxidize 1 μmol of ABTS per minute under the assay conditions.

### Optimization of enzymatic delignification of pineapple leaf waste

Parameters like solid loading (5–40% w/v), enzyme concentration (100–1000 IU/mL), incubation time (2–12 h), temperature (30–60 °C) and pH (3–10) were selected to study their effect on enzymatic delignification process and further optimization was done by RSM based on CCD. After delignification process, the solid residue was washed, dried and used for estimation of residual lignin and percentage delignification.

1 $$\% \,Delignification\,\, = \,\,\frac{Initial\,\,lignin\, - Final\,lignin}{Initial\,lignin}\,\, \times \,\,100\,\,\, \times \,\,solid\,recovery\,fraction.$$

Optimization and evaluation of enzymatic delignification of pineapple leaf waste was carried out using three-level, 2^5^ full factorial CCD with five process parameters. The boundary parameters studied in the process of enzymatic delignification were solid loading (15–25% w/v), incubation time (5–7 h), 35–45 °C, pH (6–8) and enzyme concentration (300–700 IU/mL). The experimental design and analysis of data were done using MINITAB 16 software. The designed experimental runs were tabulated in uncoded terms − 1, 0, + 1 as lowest, middle and highest level of five variables respectively in Table 1. The effect of independent factors and their interactions on % delignification was represented as polynomial quadratic regression equation. The interactive effects among various parameters was analysed based on 3D response surface plots.

**Table 1:** Experimental design for enzymatic delignification of pineapple leaf waste in terms of uncoded level of variables based on central composite design

Run order	Solid loading (% w/v)	Incubation time (h)	Temperature (°C)	pH	Enzyme concentration (IU/mL)	Delignification (%)	
						Experimental	Predicted
1	20	6	40	7	500	69.91	70.53
2	20	6	40	7	500	68.56	70.53
3	15	6	40	7	500	71.92	73.44
4	25	7	45	8	700	69.30	69.53
5	15	7	45	6	700	71.24	71.00
6	20	6	40	8	500	68.90	68.52
7	15	5	45	6	300	70.41	70.40
8	15	7	45	8	300	62.21	62.29
9	20	6	45	7	500	73.52	72.64
10	25	7	35	6	700	66.79	67.12
11	15	7	35	8	700	57.89	57.52
12	25	5	45	8	300	58.81	59.27
13	25	5	45	6	700	67.56	67.70
14	25	7	35	8	300	56.33	56.99
15	20	6	40	7	500	70.79	70.53
16	15	5	45	8	700	64.13	63.57
17	20	6	40	7	300	65.52	62.93
18	15	7	35	6	300	50.87	51.05
19	20	6	40	7	700	67.52	68.44
20	20	6	40	7	500	70.49	70.53
21	20	7	40	7	500	68.45	66.80
22	25	7	45	6	300	64.33	65.11
23	20	6	40	7	500	68.23	70.53
24	25	6	40	7	500	78.14	74.96
25	15	5	35	8	300	57.89	57.76
26	20	5	40	7	500	66.15	66.13
27	25	5	35	6	300	52.43	53.00
28	15	5	35	6	700	58.69	58.23
29	20	6	35	7	500	65.69	64.90
30	25	5	35	8	700	65.25	65.26
31	20	6	40	7	500	68.53	70.53
32	20	6	40	6	500	71.24	69.95

### Energy density measurement

The energy density of the raw and delignified pineapple leaf waste was measured using Bomb Calorimeter (Oxygen Bomb Calorimeter, Eastern Instruments, Kolkata, India). The samples were dried at 60 °C in an oven for removing the moisture and then formed into pellets. The heat content of the samples was determined in the presence of excess oxygen and high pressure (2.75 × 10^6^ Pa). The energy density (kJ/g) of the solid samples were determined as following:

2 $$Energy\,\,density\,({\text{kJ}}/{\text{g}})\,\, = \frac{{W\,\, \times \,(T_{2\,\,} - T_{1\,} \,)}}{M}$$

where W is the water equivalent of calorimeter (9.748 kJ/°C), M is the mass of the sample and (T_2_ − T_1_) is the rise in the temperature.

### Structural characterization

The morphological studies of raw and delignified pineapple leaf waste were carried out by scanning electron microscopy (SEM). The dried samples were mounted on a stub of metal with adhesive, coated with gold and viewed under JEOL JSM 5800 SEM (Jeol Ltd., Tokyo, Japan).

Fourier transform infrared spectroscopy (FTIR) was carried out for both raw and delignified samples of pineapple leaf waste using KBr pellet technique in spectral range of 400–4000 cm^−1^ with a resolution of 0.5 cm^−1^. The FTIR analysis was mainly intended to study the functional groups available in lignocellulosic substrate and changes in intensity of absorption bands due to enzymatic action.

The crystallinity of raw and delignified samples of pineapple leaf waste was determined through X-ray diffraction (XRD) studies using CoK*α* radiation (*α *= 1.79 Å) at 20 mA and 40 kV. The samples were scanned from 2*θ *= 15° to 75° with a speed of 3°/min. The percentage crystallinity was calculated from [(*I*_002_ − *I*_am_)/*I*_002_] × 100, where *I*_002_ represents maximum peak intensity (2θ) between 22° and 23° and *I*_am_ represents minimum peak intensity (2θ) between 18° and 19° for cellulose *I* [22].

### Porosity analysis

Specific surface area, pore volume and pore diameter of pineapple leaf waste sample were studied using the Brunauer–Emmett–Teller (BET) and Barrett–Joyner–Halenda (BJH) method. The adsorbate suitable for evaluation of porosity of pineapple leaf waste was nitrogen at 77 K using Quantachrome Autosorb Automated Gas Sorption System apparatus. The 0.2 mm particle sized pineapple leaf waste sample was degassed for overnight at 60 °C to remove the moisture from the pores.

## Results and discussion

### Biochemical characterization of pineapple leaf waste

Biochemical characterisation of pineapple leaf waste clearly indicate that it was rich in cellulose (42.29% ± 0.32, w/w) and hemicellulose (25.18% ± 0.25, w/w) with significant lignin (13.05% ± 0.33, w/w) content. The reported composition of cellulose (43.53% ± 1.17, w/w), hemicellulose (21.88% ± 0.22, w/w) and lignin (13.88% ± 1.70, w/w) of pineapple leaf were in close proximity with that obtained in the present study [23]. For efficient utilization of holocelluloses for biofuel production, the lignin barrier should be distorted by laccase mediated delignification process.

### Elemental analysis of pineapple leaf waste

The elemental analysis (Table 2) depicts a slight decrease in CHNS content in the delignified substrate in comparison with the raw substrate. During laccase mediated delignification, the breakdown of C=C, C–H bonds in lignin and C=O bonds in hemicellulose and lignin were observed (FTIR analysis), which might be the plausible reason for the decrease. The reduction in the carbon content of the delignified pineapple leaf waste was estimated to be around 1.8% (w/w). The diminutive decrease in the carbon content during the process proves the effectiveness of the enzymatic delignification for better fuel properties of the substrate.

**Table 2:** Elemental analysis of raw and delignified pineapple leaf waste

Substrate	C (wt%)	H (wt%)	N (wt%)	S (wt%)	O (wt%)
Raw	39.16	4.77	1.97	0.17	53.93
Pretreated	37.37	4.65	1.54	0.13	56.31

Oxygen (wt%) was calculated by difference

### Parameters affecting enzymatic delignification process

**Fig. 1:**
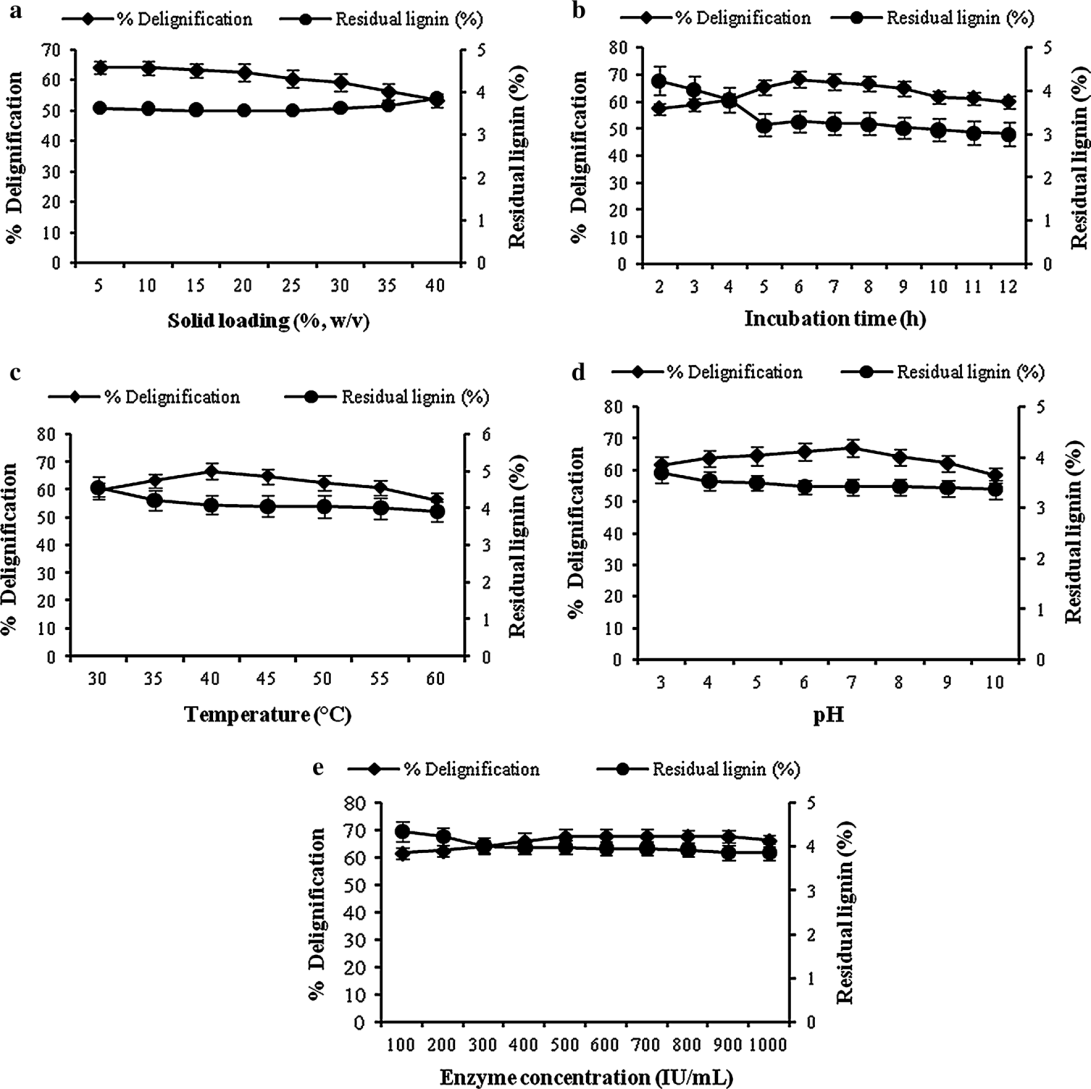
Effect of **a** solid loading **b** incubation time **c** temperature **d** pH **e** enzyme concentration on percentage delignification and residual lignin. Raw pineapple leaf waste powder is the substrate and effect of various parameters like solid loading (5–40% w/v), incubation time (2–12 h), temperature (30–60 °C), pH (3–10) and enzyme concentration (100–1000 IU/mL) on enzymatic delignification process of the substrate has been studied

A range of various parameters were considered for studying their effect on delignification of pineapple leaf waste. The percentage delignification and its corresponding residual lignin for all the parameters were shown in the Fig. 1. It was found that solid loading 20% (w/v), incubation time 6 h, 40 °C, pH 7 and enzyme concentration 500 IU/mL showed maximum percentage delignification.

### Optimization of enzymatic delignification of pineapple leaf waste

Using the experimental data of enzymatic delignification and various interaction terms of experimental variables, a second order polynomial equation was fitted for the process which is represented as:

3 $$\begin{aligned} {\text{Delignification (\% )}} & = - 337.974 - 6.487\; \times {\text{solid loading}} + 38.983 \times {\text{incubation time}} \\ & \quad + 9.811 \times {\text{ temperature }} + 31.764\; \times {\text{pH }} + 0.120 \times {\text{enzyme concentration}} \\ & \quad + 0.147 \times {\text{ solid loading }} \times {\text{ solid loading }} - 4.064 \times {\text{ incubation time }} \times {\text{ incubation time}} \\ & \quad - 0.070 \times {\text{ temperature }} \times {\text{ temperature }} - 1.294 \times {\text{ pH }} \times {\text{ pH}} \\ & \quad -0.001 \times {\text{ enzyme concentration }} \times {\text{ enzyme concentration}} \\ & \quad + \;0.270 \times {\text{ solid loading }} \times {\text{ incubation time}} - 0.059 \times {\text{ solid loading }} \times {\text{ temperature}} \\ & \quad + \;0.096 \times {\text{ solid loading }} \times {\text{ pH }} + 0.002 \times {\text{ solid loading }} \times {\text{ enzyme concentration}} \\ & \quad + \;0.107 \times {\text{ incubation time }} \times {\text{ temperature }} - 0.281 \times {\text{ incubation time}} \\ & \quad \times {\text{ pH }} + 0.005 \times {\text{ incubation time }} \times {\text{ enzyme concentration}} \\ & \quad - 0.346 \times {\text{ temperature }} \times {\text{ pH}} - 0.001 \times {\text{ temperature }} \times {\text{ enzyme concentration}} \\ & \quad - \; 0.002 \times {\text{ pH }} \times {\text{ enzyme concentration}}. \\ \end{aligned}$$

In the above equation, units for solid loading, incubation time, temperature and enzyme concentration are % (w/v), h, °C and IU/mL respectively. Analysis of variance (ANOVA) of the quadratic equation for the delignification (%) of pineapple leaf waste has been summarized in Table 3. The regression model for enzymatic delignification of pineapple leaf waste showed a considerable high *F* value (27.23) and very low *P* value (< 0.001) thus specifying the significance of the model. High R^2^ value (0.96) of laccase delignified pineapple leaf waste demonstrates the robustness of the model. No significant difference between the R^2^ and adjusted R^2^ values was observed, indicating that the regression model is reliable and competent for the process. It is noted from the coefficients of terms in the regression equation that the individual terms of incubation time, pH, temperature and square terms of pH have profound influence on the percentage of delignification.

**Table 3:** ANOVA analysis of RSM model for enzymatic delignification of pineapple leaf waste

Source	DF^a^	Seq SS^b^	Adj SS^b^	Adj MS^c^	*F*	*P*
Regression	20	1142.52	1142.52	57.126	14.95	< 0.001
Linear	5	427.90	152.03	30.406	7.96	0.002
Square	5	520.36	520.36	104.072	27.23	< 0.001
Interaction	10	194.26	194.26	19.426	5.08	0.006
Residual error	11	42.04	42.04	3.822		
Lack-of-fit	6	35.83	35.83	5.972	4.81	0.053
Pure error	5	6.21	6.21	1.242		
Total	31	1184.56				

R^2^ = 96.45%

R^2^(adj) = 90.00%

^a^ Degrees of Freedom

^b^ Sum of Squares

^c^ Mean Square

**Fig. 2:**
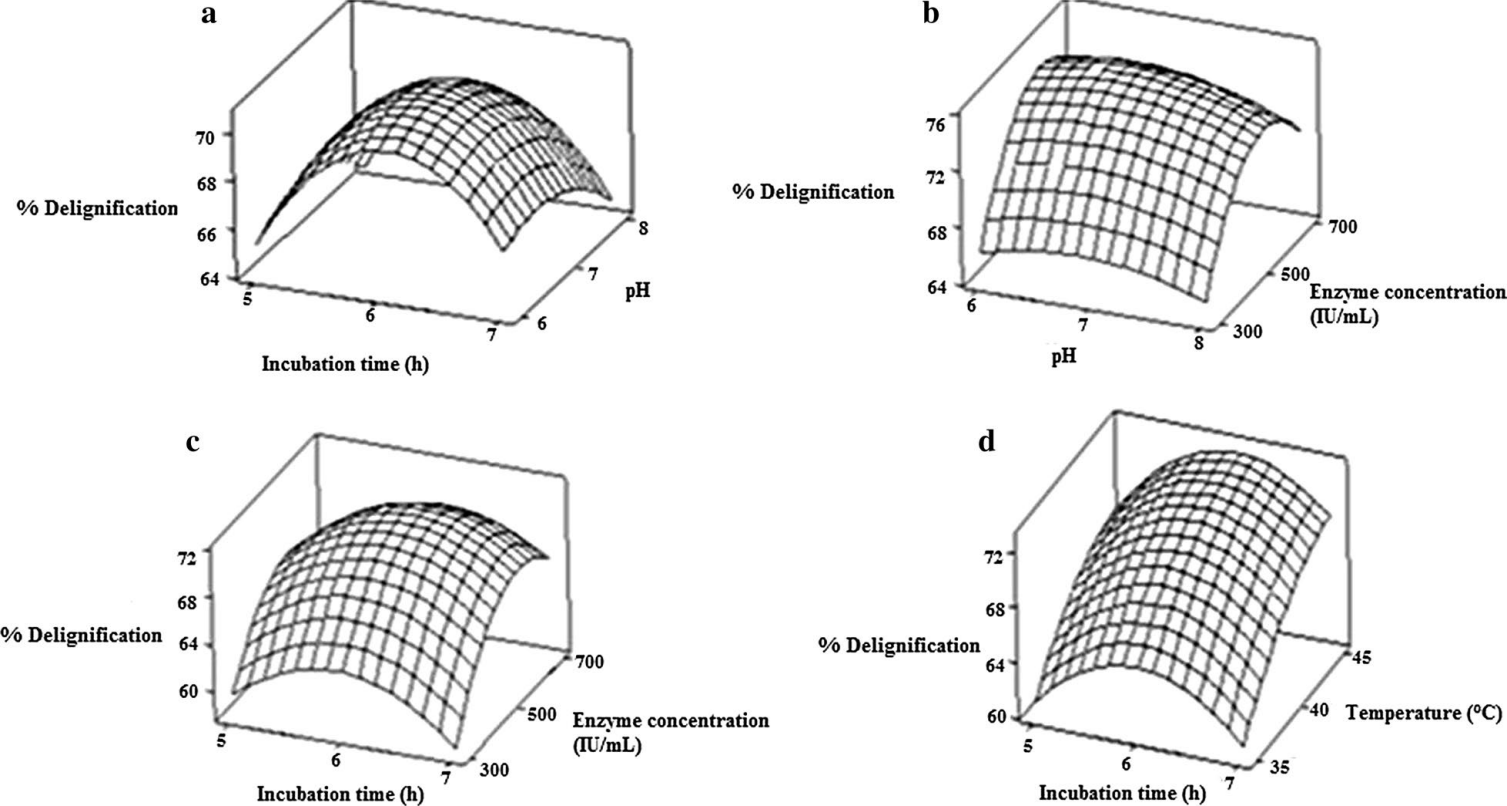
Response surface plots representing relationship between % delignification and **a** incubation time and pH **b** pH and enzyme concentration **c** incubation time and enzyme concentration **d** incubation time and temperature. Raw pineapple leaf waste powder is the substrate. Effect of interaction between two variables such as incubation time (5–7 h) and pH (6–8), enzyme concentration (300–700 IU/mL) and pH (6–8), incubation time (5–7 h) and enzyme concentration (300–700 IU/mL), incubation time (5–7 h) and temperature (35–45 °C) on the delignification (%) has been studied

The 3D response surface plots are graphical representation of the regression equation. The response surface plots pertaining to the conditions for enzymatic delignification of pineapple leaf waste were shown in Fig. 2. Each plot in the figure represents the effect of two variables on the percentage delignification. The interaction between incubation time and pH, enzyme concentration and pH, incubation time and enzyme concentration, incubation time and temperature on delignification (%) were shown in the Fig. 2a–d respectively.

Figure 2a depicts that with an increase in incubation time and pH the percentage delignification increases up to an optimum incubation time (6 h) and pH (7). Thereafter, a slight decrease was observed which may be due to the loss of enzyme stability at high pH and prolonged incubation time. From the Fig. 2b, c it was observed that percentage delignification increased up to optimum enzyme concentration (500 IU/mL), incubation time (6 h) and pH (7). After that, slight decrease in delignification percentage was observed which may be due to saturation of the catalytic sites of the enzyme or loss of stability of enzyme. Figure 2d illustrates that maximum delignification was obtained at interaction between upper limit of temperature (45 °C) and middle level of incubation time (6 h). This possibly signifies negligible thermal degradation of enzyme up to the maximum temperature level.

From the analysis of response surface plots, the optimum conditions for enzymatic delignification were found to be solid loading 25% (w/v), incubation time 5.30 h, 45 °C, pH 6.16, enzyme concentration 582.82 (IU/mL). Under these optimum conditions, the predicted delignification was 77.56% which was close to the experimental delignification 78.57%. Similar results were reported in *Bambusa bambos* where the maximum delignification of 84% was obtained at pH 6.9, 35 °C, liquid:solid ratio 6:1, incubation time 8 h, and enzyme concentration 400 IU/mL [14]. It is not essential to degrade the entire lignin content of lignocellulosics to obtain a significant increase in digestibility, rather 20–65% of the lignin degradation can significantly improve the yield of enzymatic hydrolysis depending on the source of cellulose [24]. Through RSM based on CCD, maximum delignification of pineapple leaf waste was obtained at high solid loading and short incubation time, which makes the process viable.

### Energy density measurement

Energy density plays a very important role not only in determining the efficacy of lignocellulosic feedstock as a potential biofuel producer but also for validating the efficiency of the process involved in the biofuel generation viz. the delignification process. A low energy density feed stock is less energy efficient to convert into biofuel than that of high energy density feedstock. The energy density of the raw and enzymatically delignified pineapple leaf waste was found to be 17.13 ± 0.65 kJ/g and 15.95 ± 0.5 kJ/g respectively. The reduction in the energy density of delignified pineapple leaf waste compared to the raw substrate may be due to lignin degradation which is in accordance with the reported work where energy density of the lignocellulosic feedstock (Kans grass) was reduced after enzymatic delignification process [25]. There exists a direct relationship between the lignin content and the higher heating value (HHV) of the biomass. The heating value of lignin is comparatively high than that of cellulose as the degree of oxidation is higher for lignin [26].

### Structural characterization of delignified pineapple leaf waste

**Fig. 3:**
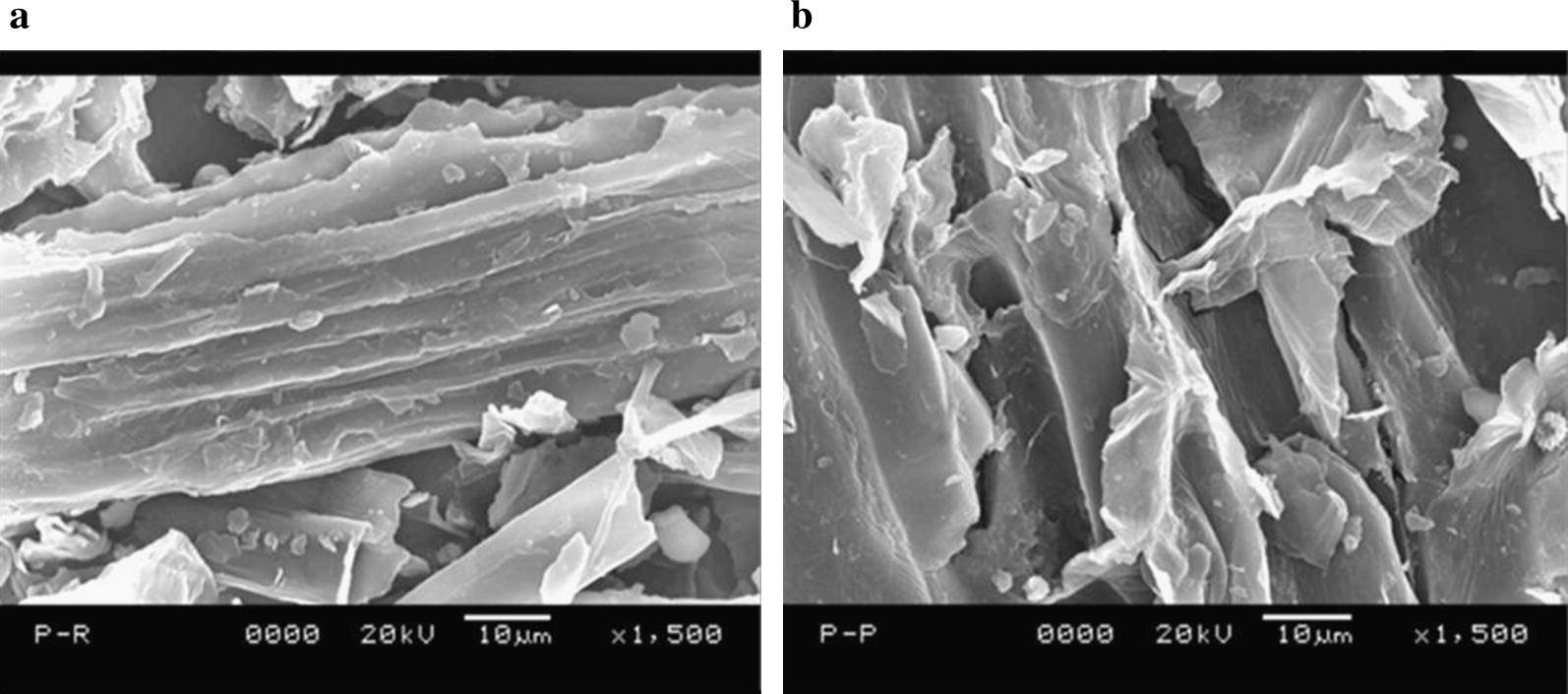
Scanning electron microscopy images of **a** raw and **b** delignified pineapple leaf waste. Maximum delignification of pineapple leaf waste has been obtained at solid loading 25% (w/v), incubation time 5.30 h, 45 °C, pH 6.16 and enzyme concentration 582.82 (IU/mL). The structural morphology of raw and delignified substrate has been studied

The changes in structure of pineapple leaf waste after enzymatic delignification under optimum conditions from RSM were assessed through SEM studies. The delignification process creates cracks and pores on the substrate due to the removal of lignin which was clearly observed in the SEM images of raw and delignified biomass (Fig. 3). The delignification process makes the lignocellulosic substrate more susceptible to saccharification by exposing the cellulosic material to cellulase and thereby enhances the yield of fermentable sugars.

**Fig. 4:**
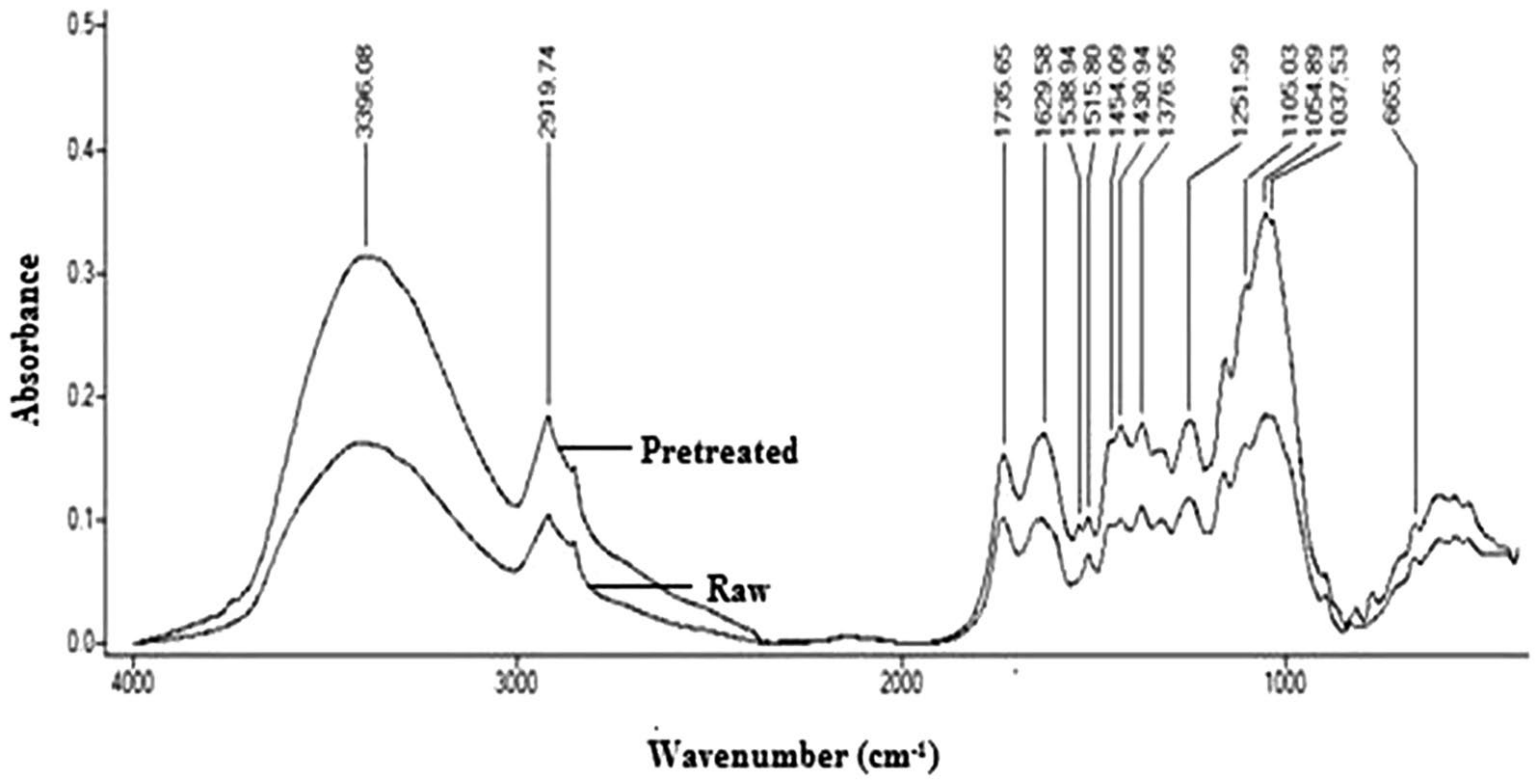
Fourier transform infrared spectra of raw and delignified pineapple leaf waste. Maximum delignification of pineapple leaf waste has been obtained at solid loading 25% (w/v), incubation time 5.30 h, 45 °C, pH 6.16 and enzyme concentration 582.82 (IU/mL). Changes of the functional groups and compositional modifications in laccase delignified substrate against the raw substrate have been studied

FTIR has been used to explore the changes of the functional groups and compositional modifications in laccase delignified substrate against the raw substrate with significant changes in the absorption spectrum of delignified substrate (Fig. 4). Notable decrease in the absorption peaks was observed at 3396 cm^−1^ (OH stretching of lignin), 2919 cm^−1^ (C–H stretching of lignin), 1735 cm^−1^ (C=O stretching of hemicellulose), 1629 cm^−1^ (C=O stretching vibration in conjugated carbonyl of lignin), 1538 cm^−1^ and 1515 cm^−1^ (C=C stretching vibrations of aromatic rings of lignin), 1454 cm^−1^ (aliphatic part of lignin), 1430 cm^−1^ (C–H in-plane deformation of lignin), 1376 cm^−1^ (aliphatic C–H stretching in methyl and phenol alcohol) 1251 cm^−1^ (syringyl ring breathing and C–O stretching in lignin and xylan), 1050 - 1150 cm^−1^ (C–OH stretching vibration of the cellulose and hemicelluloses), 1037 cm^−1^ (C–O, C=C and C–C–O stretching) and 665 cm^−1^ (aromatic C–H bending of lignin) after enzymatic delignification process [27–29]. The prime reason for the decrease in these absorption peaks is the cleavage of the lignin side chains by laccase without much structural alterations of cellulose and hemicellulose [30].

**Fig. 5:**
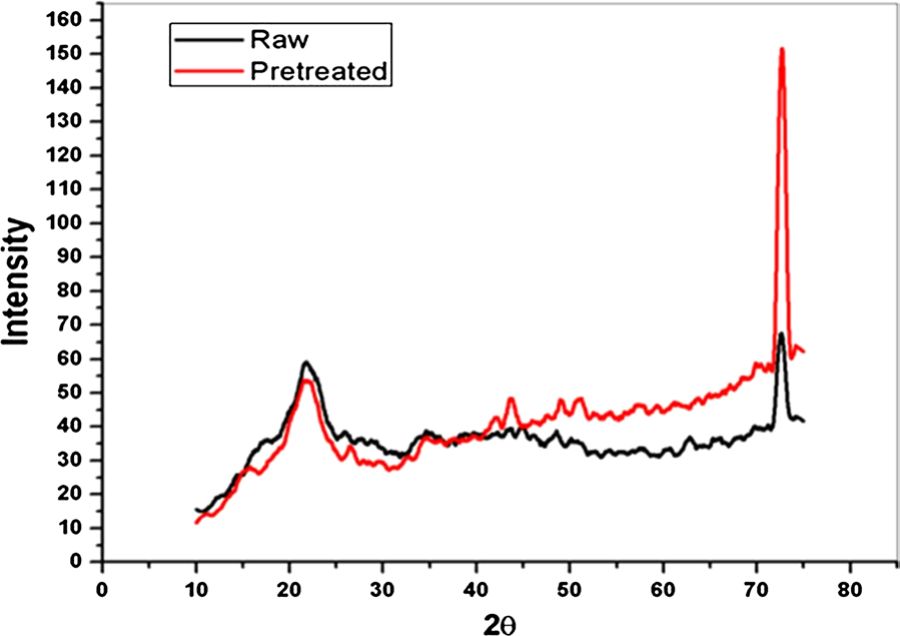
X-ray diffraction spectra of raw and delignified pineapple leaf waste. Maximum delignification of pineapple leaf waste has been obtained at solid loading 25% (w/v), incubation time 5.30 h, 45 °C, pH 6.16 and enzyme concentration 582.82 (IU/mL). Change in cellulose crystallinity of laccase delignified substrate has been studied against the raw substrate

The cellulose accessibility to cellulase for efficient saccharification is governed by various factors like cellulose crystallinity, lignin and hemicellulose content, its distribution in the substrate and porosity [31]. XRD provides an accurate measure of the crystallinity of cellulose. It was observed (Fig. 5) that cellulose crystallinity of raw and delignified substrates were 27.50% and 33.96% respectively indicating an increase in relative crystallinity by 6.46% which may be due to the removal of lignin from pretreated substrate [32]. These results are similar to those obtained in case of *Lantana camara* where an increase in crystallinity of enzymatically delignified sample (25.21%) was observed over that of the raw sample (19.57%) [32].

The cellulose, hemicellulose and lignin contents of raw and delignified pineapple leaf waste under optimized conditions are provided in Table 4. A significant decrease in lignin (4.66-fold) and hemicellulose content (1.31-fold) was observed from delignified substrate as compared to the raw substrate. The cellulose obtained from pineapple leaf waste after delignification process is nearly 1.09-folds higher than that obtained from the raw pineapple leaf waste. These biochemical changes illustrate the action of laccase on pineapple leaf waste.

**Table 4:** Biochemical composition of raw and delignified pineapple leaf waste

Component	Initial	@5.30 h
Lignin (%, w/w)	13.05 ± 0.33	2.78 ± 0.25
Cellulose (%, w/w)	42.29 ± 0.32	45.20 ± 0.98
Hemicellulose (%, w/w)	25.18 ± 0.25	19.80 ± 0.73

To determine the effect of delignification on production of reducing sugar, saccharification was performed with raw and delignified pineapple leaf waste. The reducing sugar (mg/g) from raw and delignified substrates was found to be 189.0 ± 8.89 and 492.33 ± 3.1 respectively with a significant increase of 2.6-folds. The results show that enzymatically delignified pineapple leaf waste can produce considerably high amount of reducing sugar after saccharification.

### Measurement of specific surface area, pore size and pore volume of raw and delignified substrate

One of the important parameters that affect the hydrolysis rate of cellulose is specific surface area. The absorption of cellulase on the surface of cellulose is mandatory for the hydrolysis reaction to occur as the extent of hydrolysis depends on the surface area accessible to the enzymes. Based on BET analysis (Table 5), surface area of raw and delignified pineapple leaf waste under optimized conditions were found to be 1.41 and 2.51 m^2^/g respectively, that showed an increase in the surface area by 1.78-folds. The obtained results from the present study are substantially higher than the reported work where surface area analysis of raw and ionic liquid pretreated oil palm empty fruit bunch was 0.55 m^2^/g and 0.75 m^2^/g [33]. BJH analysis was carried out to determine the pore size and its volume through N_2_ adsorption and desorption techniques.

**Table 5:** Changes in surface area, pore volume and pore diameter of pineapple leaf waste before and after enzymatic delignification

Sample	Surface area (m^2^/g)	Pore volume (cc/g)	Pore diameter (nm)
Raw	1.41	4.26 × 10^−3^	12.00
Delignified	2.51	5.51 × 10^−3^	13.95

There exist a direct relation between the pore volume or interior surface area of the substrate and the extent of hydrolysis by cellulase [34]. Enhanced cellulose hydrolysis was observed when the pores of the substrate were large enough to accommodate adequate cellulase [35]. The rate-limiting pore size for the hydrolysis of lignocellulosic substrates was reported to be 5.1 nm [36]. The pore size (diameter) of raw and delignified pineapple leaf waste from BJH analysis was found to be 12.00 nm and 13.95 nm respectively which suggests the pineapple leaf waste to be mesoporous in nature. Laccase delignified pineapple leaf waste with increased pore volume (by nearly 1.3-folds, Table 5) can improve the production of reducing sugar.

The prime objective of the delignification process is to remove lignin that acts as barrier to cellulose which is major contributor of reducing sugar. Conventional pretreatment process not only leads to a significant amount of hemicellulose loss along with lignin but also results in formation of intermediates such as furfurals and hydroxymethylfurfurals that may act as inhibitors for the fermentation process. Besides, conventional pretreatment processes are also associated with equipment corrosion, high cost, high water requirement, excess use of chemicals, formation and incorporation of salts into the biomass and enormous waste production. In order to overcome these issues, enzymatic delignification of biomass was carried out in the present study. Enzymatic delignification is an eco-friendly process which can work under mild operating conditions. Laccase being specific, acts on lignin moieties of the substrate and has less effect on the structure and composition of cellulose and hemicelluloses. Laccase mediated delignification enhances saccharification efficiency of cellulase thereby leading towards cleaner production of biofuel [37] such as biobutanol, bioethanol and bio-chemicals like lactic acid, acetic acid, sorbitol, xylitol, vanillin etc.

## Conclusion

Biochemical characterisation of pineapple leaf waste shows the presence of high cellulose and hemicellulose with significant lignin content. Laccase mediated delignification process degrades lignin up to 78.57% (w/w) which is accompanied by an increase in cellulose crystallinity of 6.46%. This substantiates the effective action of laccase on pineapple leaf waste during delignification process which results in increased production of reducing sugar (492.33 ± 3.1 mg/g). Structural characterization as well as surface area and porosity analysis of the substrate further corroborate the enzymatic delignification process. The energy density studies of pineapple leaf waste prove that it is a competent substrate for biofuel production. In the perspective of cleaner production, enzymatic delignification of biomass is a green, sustainable and eco-friendly process for improved production of reducing sugar that contributes for efficient biofuel production. The agro-industrial waste utilization for generation of value added products is not only an effective way of waste removal from the environment but also economical.

## Abbreviations

RSM: response surface methodology
CCD: central composite design
FTIR: Fourier transformed infrared spectroscopy
XRD: X-ray diffraction
SEM: scanning electron microscopy
Mt: million tonnes
Mha: million hectares
ABTS: 2,2′-azino-bis (3-ethylbenzothiazoline-6-sulphonic acid
IU: international units
kJ/g: kilojoules per gram
nm: nanometre
cc/g: cubic centimetre per gram
mg/g: milligram per gram
